# Apes in Space: Saving an Imperilled Orangutan Population in Sumatra

**DOI:** 10.1371/journal.pone.0017210

**Published:** 2011-02-16

**Authors:** Gail Campbell-Smith, Miran Campbell-Smith, Ian Singleton, Matthew Linkie

**Affiliations:** 1 Durrell Institute of Conservation and Ecology, University of Kent, Canterbury, Kent, United Kingdom; 2 Orangutan Information Centre, Human-Orangutan Conflict and Mitigation Programme, Medan, North Sumatra, Indonesia; 3 Sumatran Orangutan Conservation Programme, PanEco Foundation, Medan, North Sumatra, Indonesia; 4 Fauna & Flora International, Cambridge, United Kingdom; University of Maribor, Slovenia

## Abstract

Deforestation rates in Sumatra are amongst the highest in the tropics. Lowland forests, which support the highest densities of orangutans, are particularly vulnerable to clearance and fragmentation because they are highly accessible. Consequently, many orangutans will, in the future, live in strictly or partially isolated populations. Whilst orangutans have been extensively studied in primary forests, their response to living in human-dominated landscapes remains poorly known, despite it being essential for their future management. Here, we focus on an isolated group of critically endangered Sumatran orangutans (*Pongo abelii*) that co-exist with farmers in a mixed agroforest system consisting of degraded natural forest, smallholder (predominantly rubber) farms and oil palm plantations. Over 24 months we conducted the first ever spatial assessment of orangutan habitat use in the human-transformed landscape of Batang Serangan, North Sumatra. From 1,204 independent crop-raiding incidents recorded, orangutans showed strong foraging preference for mixed farmland/degraded forest habitat over oil palm patches. The core home range areas of the eight adult orangutans encompassed only 14% of the available study area. Monthly home range sizes averaged 423 ha (±139, SD) for males, and 131±46 ha for females, and were positively influenced by wild and cultivated fruit presence, and by crop consumption. The average daily distance travelled was similar for both adult males (868 m±350, SD) and females (866 m±195), but increased when orangutans raided crops. These findings show that orangutans can survive, demographically, in certain types of degraded landscapes, foraging on a mixture of crops and wild fruits. However, the poor quality habitat offered to orangutans by oil palm plantations, in terms of low food availability and as a barrier to female movements, is cause for concern since this is the land use type that is most rapidly replacing the preferred forest habitat across both Sumatran and Bornean orangutan ranges.

## Introduction

As human populations increasingly encroach upon natural habitats, conflicts between people and wildlife are inevitable due to competition for space and resources [1]. As both primary and secondary forests are converted to agriculture, forest-dwelling species may shift towards exploiting human settlements and fields to supplement a dwindling supply of wild foods or take advantage of nutritious crops that seasonally ripen in abundance [2]. Those wildlife species that can adapt to marginal human-dominated habitats may become pests and be persecuted as a consequence.

Previous research suggests that many factors can influence the temporal characteristics of crop-raiding by large mammals. For example, crop-raiding incidents of four mammal species in Indonesia were positively correlated with higher rainfall [3], and incidents involving elephants in India were strongly related to natural migratory and dispersal behaviours [4]. Spatial patterns of crop-raiding have also been explained by factors such as the availability and distribution of water [5], the number of and distance to neighbouring farmlands [6] and forest-agricultural margins [6], [7]. In Kibale National Park, Uganda, 90% of crop-damage occurred close to the forest edge [8], but different crop-raiding species may travel different distances from a forest boundary into neighbouring farmlands [6], [7], [8], [9]. Other spatial factors also come into play, such as what types of barrier exist (e.g. rivers and roads) between forest and farmland [10], the patterns of cultivation [11], levels of human activity [12] and preferences for particular crops [13]. Indeed, the sheer variety and complexity of factors make it difficult for farmers to protect crops from raiding, and some attempt to address this by planting buffer crops near the forest boundary, to reduce the economic impact of losing their main cash crop and to reduce the investment required in crop guarding [14], [15], [16].

Non-human primates, such as *Macaca* sp. in Asia, and *Papio* sp. and *Cercopithecus* sp. in Africa, are considered particularly problematic as crop-raiders [14], [17]. With a few exceptions [6], [14], [18], [19], little has been written about patterns of crop-raiding by great apes. More specifically, conflict between Sumatran orangutans (*Pongo abelii*) and people is emerging as an important issue [20], [21] that is predicted to dramatically increase given the alarming rates of deforestation, predominantly caused by oil palm expansion in Indonesia [22]. Such conflict may result in damage to economically valuable crops such as oil palm (*Elaeis guineensis*), rubber tree (*Hevea brasiliensis*) and sugarcane (*Saccharum officinarum*) [23]. It may also result in a significant shift in orangutan diets, from wild species to greater reliance on cultivated crops, and consequently influence ranging behaviour and the management strategies adopted for mitigating conflicts. Sumatran orangutans are strictly protected under Indonesian law and cannot be legally managed in the same way that other, less protected ‘pest’ species often are (i.e. shot or otherwise removed). With communities unable to intervene in this way, according to the law, human orangutan conflicts can lead to considerable confusion and frustration and, ultimately, serious resentment of the species among local communities, which in turn can lead to their elimination too, albeit surreptitiously and illegally.

Essentially, there is no scientific literature documenting patterns of crop-raiding by orangutans or indeed their habitat use within human-dominated landscapes. This paper aims to address this knowledge gap and conservation imperative by: i) investigating crop-raiding patterns in oil palm plantations and mixed farmland/degraded forests; and, ii) investigating the influence of ecological variables and crop-raiding patterns on orangutan day journey length and home range sizes.

## Materials and Methods

### Ethics Statement

All research protocols applied within this manuscript were reviewed and approved by the University of Kent and the Indonesian Ministry of Forestry (permit # 1039/FRP/SM/V/2007 and # 2756/FRP/SM/XI/2008) and adhered to the Principles for the Ethical Treatment of Non-Human Primates and to Indonesian law.

### Study Area

**Figure 1:**
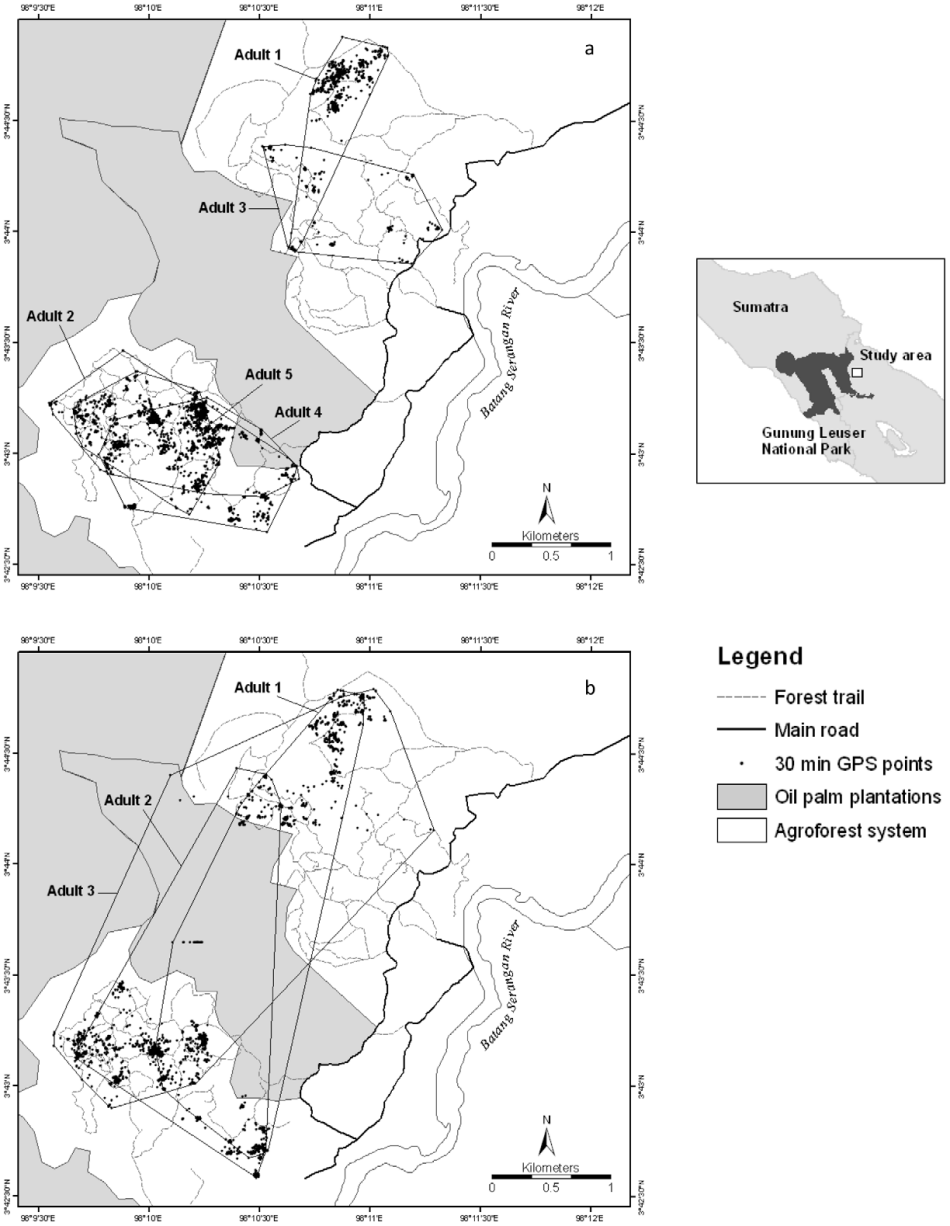
Orangutan home range patterns for (a) five adult females (2936 data points) and (b) three adult males (2034 data points) in Batang Serangan, North Sumatra.

The Batang Serangan study area, in Langkat district, North Sumatra, (3° 43′58.99"N; 98° 11′41.99"E) is 25 kilometers from the Gunung Leuser National Park; a stronghold for the Sumatran orangutan. Batang Serangan offers a rare opportunity to determine patterns of crop-raiding and movement patterns by orangutans that are completely isolated within a human-disturbed landscape (Fig. 1). The 3,234 ha closed agroforest system varies in altitude from 28–165 m asl and contains remnants of old growth degraded forest, intermixed with cultivated crops, especially the main cash crops of oil palm and rubber, and to a lesser extent subsistence fruit tree crops, such as jackfruit (*Artocarpus integer*), durian (*Durio zibethinus*), jengkol (*Archidendron pauciflorum*), and petai (*Parkia speciosa*). Such small-scale intercropping has been a constant feature of the area for over three decades. This mixed farm area is completely bordered and partially bisected by commercial monoculture oil palm plantations, consisting of mature palms.

Batang Serangan currently supports 16 known and habituated individual orangutans [21], which would have once been part of a larger population that occupied the wider landscape before the natural forests were cleared for agriculture.

### Field Surveys

All field data were collected from February 2007 to February 2009. The number of crop-raiding incidents was recorded through daily farm visits by nine enumerators. An independent crop-raiding incident was used as the basic unit of measurement, whereby crop-raiding by an individual orangutan on the same farm, on the same day, was classified as a single event, irrespective of whether it raided more than once. This unit calculation was modified from that developed and applied for measuring wildlife crop damage in Uganda [14]. The datasheet format used for recording orangutan crop-raiding was modified from that developed by the IUCN/SSC African Elephant Specialist Group for monitoring human-elephant conflict [24]. When a crop-raiding incident was reported, its location was recorded using a global positioning system (GPS) unit.

The home range sizes of five adult female and three adult male orangutans were determined through individual focal animal follows [25]. Once a focal orangutan was identified, the three followers undertook, when possible, nest-to-nest follows for a maximum of five consecutive days unless the individual was lost by the surveyor. Data were recorded on the individual's (GPS) position every 30 minutes and the individual's behaviour at 2-min intervals from exiting its nest in the morning to settling in its night nest that evening. For focal sampling data, four main activities (travelling, resting, feeding and other) were recorded. Feeding data were collected for food types (cultivated and wild) and recorded as; fruit, leaves (differentiating between young and old), seed (no flesh consumed), bark (inner cambium and phloem) and branch (fibres; [25]). From these data, orangutan crop-raiding days and non-crop-raiding days (i.e. when only wild fruit was consumed) were identified.

On a daily basis the smallholder farmlands were monitored for fruit availability by at least two observers, using binoculars. Cultivated crop (hereafter ‘fruit’) and wild fruits (unripe and ripe) were recorded as ‘present’ on a particular farm if at least five tree species had fruits growing in the majority (i.e. >50%) of their individual canopies. On the landscape level, these data were compiled to determine which species were available that month based on at least ten farms having the fruiting species recorded as ‘present’.

### Data Analysis

All GPS coordinates were entered into ArcGIS v9.2 software (ESRI Inc., Redlands CA, USA) to determine crop-raiding locations, home range sizes and day journey lengths. The resulting data, including information on whether it related to crop-raiding or non crop-raiding days, were then imported into SPSS v16.0 software (SPSS, Chicago, USA) for further analysis. Continuous data were logarithmically transformed to reduce the disproportionate influence of outliers. Collinearity between independent variables was tested (Pearson's rank correlation coefficient, r), but none found.

Spatial patterns of crop-raiding were examined by superimposing a 100 m×100 m grid across the recorded orangutan range within the study area [26]. Next, 100 grid cells were randomly selected (50 cells in oil palm and 50 cells in mixed agroforest habitat types), with the condition that cells be at least 200 m apart to minimise spatial autocorrelation. The number of crop-raiding incidents was then extracted for each of the 100 grid cells, along with information on mean elevation and distance to nearest village, which were closely related to roads. Logistic regression models were then used to determine which combination of spatial factors best explained the presence or absence of crop-raiding in both oil palm and mixed agroforest habitat types. The final model was selected based on its delta Akaike Information Criterion (ΔAIC) values and Akaike weights (w_i_). The presence of spatial autocorrelation was tested by calculating Moran's *I* statistic using Crime-Stat v3.2 software (N. Levine and Associates, Annadale, VA, USA).

To maximise the dataset, all ranging data from follows ≥3 hours were included in the analysis of home range sizes [27]. Home range sizes were calculated using the three methods commonly used in non-human primate studies [26], [28], [29], [30]: a minimum convex polygon (MCP) method; a 100 m×100 m resolution grid cell-based method; and, a fixed kernel density estimation (KDE) method, taken at the 95% and 50% values. These methods were selected as they each have their own unique merits, but they also have limitations, and home range estimates can be highly sensitive to sample size. The MCP method may overestimate home range size since the vector polygon is evaluated from the outermost points, possibly including areas that are not used, or may underestimate home ranges if coverage is incomplete (both spatially and temporally). The grid cell method may underestimate home range size if only a single GPS coordinate is registered per day or overestimate home range size if only a small proportion of the entire grid cell is surveyed or used by the animal. The KDE is regarded as a more robust technique and is widely applied in quantifying animal range use, although it has rarely been used for orangutans [31]. For these reasons, to enable direct comparisons with other orangutan studies, only the results from the MCP method were used in additional statistical analyses. As well as individual range sizes, range overlap between individuals was calculated as the intersection between respective annual ranges using MCP data using the intersect method in Analysis tools of ArcGIS. The home range size of each individual orangutan was estimated on a monthly basis and compared between males and females (ANOVA). Orangutan core areas (defined as the continuous areas in which an individual spends a high proportion of its time) were identified using the KDE at 50% values, the most suitable method.

Day journey lengths were measured by programming all GPS units to automatically record coordinates continuously throughout the day, whenever satellite coverage permitted. Only GPS track logs collected during full day follows (n = 157) were used. Track logs were linked to the focal animal observations undertaken at 2-minute intervals allowing GPS coordinates to be extracted for only those times when the animal was actually recorded as moving. This allowed all track log data to be deleted for periods when the focal was clearly not travelling, thereby reducing ‘noise’ created by field staff independently moving (e.g. to get a better view of an orangutan). Day journey lengths were calculated for each individual orangutan by entering these co-ordinates in ArcGIS and converting point data to a track line using the Hawth's Tools Animal Movement extension. The ‘daily linear distance’ (a straight-line from night nest to night nest) was also measured for each focal individual, from full day follows data. General linear models (GLM) and linear mixed-effect models were used to investigate the effects of one ecological variable (number of available wild and cultivated fruit species present per month), and one behavioural variable (crop-raiding patterns; crop-raiding/non crop-raiding days), on orangutan mean day journey length and home range size, both for the population as a whole and for individual animals.

## Results

### Crop-raiding patterns

From 706 field days, a total of 1,204 independent crop-raiding incidents were recorded on farms. These resulted in damage to 7,699 individual cultivated fruits (from 12 species) in 273 farms. From 137 crop-raiding data points within the 100 grid cell subset, the majority (96%) occurred in agroforest patches and only 4% in the oil palm patches. From the five models identified (Table 1), the summed model weights for each factor with respect to crop-raiding were habitat type (100%), elevation (97%) and distance to nearest village (29%). From the final model (#1.1), the number of crop-raiding incidents within cells covering the agroforest patches was found to be significantly higher than in cells located over oil palm patches, at lower elevations, and was not affected by spatial autocorrelation (Moran's I = −0.01, P>0.1).

**Table 1:** Best logistic regression models explaining the relationship between habitat use and the presence or absence of crop-raiding by orangutans.

Model	2log likelihood	K	ΔAIC	w*_i_*
1.1. Habitat type + elevation	68.64	3	0.00	0.692
1.2. Habitat type + elevation + distance to nearest village	68.46	4	1.82	0.279
1.3. Habitat type	77.88	2	7.24	0.019
1.4. Habitat type + distance to nearest village	76.99	3	8.35	0.011
1.5. Elevation	88.82	2	18.18	0.000

2log likelihood is the overall fit of each model, *K* is the number of parameters in each model, ΔAIC is the measurement of each model relative to the top ranked model, and w*_i_* is the AIC model weight.

### Home range patterns

A total of 4970 GPS data points were recorded at 30 min intervals during follows of eight focal animals (five adult females and their infants, and three adult males, of which two were flanged and one unflanged). The five females were sub-divided into two distinct groups by the central oil palm plantation, three on the south-west side and two on the north-eastern side, with no females recorded crossing this plantation (Fig. 1a). All of the adult males used home ranges that included both sides of the plantation, and were recorded crossing over (Fig. 1b). The degree of range overlap between the two adult females in the north-east was 17%, whilst for the three south-western females it was 90%. Range overlap for the males was similarly high, at 89%. Even though male home ranges overlapped extensively, no two males were ever recorded during focal follows to be in the same area on the same day, whereas the females would on occasions be seen in the same areas at the same time. The core areas of all orangutans were contained within a relatively small part (14%) of the study area.

Comparing the mean monthly home range estimates produced using the MCP method, showed that the three males had significantly larger home ranges than those of the five females (ANOVA, F_1,7_ = 15.000, P<0.05, Table 2). Mean monthly home range size for both male and females was larger when only data recorded on crop-raiding days were used (416 ha and 104 ha, respectively) in comparison to non crop-raiding days (179 ha and 80 ha, respectively). The GLM model (F_1,23_ = 11.010, P<0.001, adjusted r^2^ = 0.76) revealed that mean monthly home range sizes for the population were significantly influenced by the combined presence of both wild and cultivated fruits in the farms (F_1,23_ = 12.712, P<0.01) and when orangutans were crop-raiding (F_1,23_ = 4.430, P<0.05), but not by the interaction between these two factors (F_1,23_ = 0.402, P = 0.534) or days when only wild fruits were consumed (F_1,23_ = 1.025, P = 0.326).

**Table 2:** Orangutan home range (HR) size estimates (in ha) during crop-raiding (CR) and non crop-raiding (NCR) periods using minimum convex polygon (MCP), 100×100 m grid-cell based and Kernel Density Estimation (KDE) methods, and mean daily journey length (MDJ, in m; ±SD) and mean daily linear distance (MLD, in m; ±SD).

Age/sex	# follow days	Maximum HR						# nest-nest	Total	
class	(# of data points)	MCP (100%)	Grid	KDE (95%)	KDE (50%)	CR* MCP	NCR* MCP	follow days	MDJ	MLD
Adult1 ♀	29 (533)	58	123	125	233	58	15	16	780 (±637)	278 (±232)
Adult2 ♀	43 (795)	137	211	267	265	137	49	23	840 (±605)	249 (±189)
Adult3 ♀	16 (215)	187	145	286	310	104	157	13	926 (±852)	486 (±584)
Adult4 ♀	54 (1001)	142	184	121	190	128	90	38	627 (±911)	174 (±263)
Adult5 ♀	22 (392)	131	162	274	278	92	88	11	1155 (±711)	166 (±137)
Adult1 ♂	34 (570)	355	175	385	510	343	293	12	479 (±335)	224 (±190)
Adult2 ♂	26 (525)	330	135	385	353	326	46	15	968 (±712)	305 (±318)
Adult3 ♂	48 (939)	583	298	394	447	581	198	29	1157 (±706)	324 (±328)
Mean adult ♀		131	165	214	255	104	80		866 (±195)	271 (±130)
Mean adult ♂		423	203	388	437	416	179		868 (±350)	284 (±53)

* CR: during days when orangutans were recorded to raid cultivated crops, NCR: days orangutans were recorded to eat wild fruits only (i.e. not crop-raiding).

MDJ and MLD are based on full day follows (n = 157 days).

Similarly, a linear mixed-effect model revealed that individual orangutan home range size was significantly influenced by the number of wild and cultivated fruit species available (F_1,30_ = 6.070, P<0.01) and crop-raiding days (F_1,30_ = 14.256 P<0.001), but also by the interaction between these two factors (F_1,30_ = 5.735, P<0.05), but not by sex class (F_1,30_ = 0.104, P = 0.749) or when data recorded on non crop-raiding days were used (F_1,30_ = 3.665, P = 0.065). Overall, orangutans moved further across the landscape when more food was available and on the days that they ate cultivated fruits.

### Journey length patterns

The mean day journey length travelled by eight adult orangutans showed considerable variation (Table 2). On average, male orangutans travelled 868 m per day (±350 SD) and females 866 m (±195). There was no significant correlation between an orangutan's monthly home range size and their mean day journey length per month (r = −0.152, P = 0.488). As would be expected in a restricted habitat (and the limitations it imposes on how far the animals can travel in any one direction) the mean day journey length and the mean linear distance travelled per month were correlated (r = 0.740, P<0.01) and, so, just the former was used in subsequent analyses. The GLM model (F_1,23_ = 4.724, P<0.01, adjusted r^2^ = 0.51) revealed that the mean day journey length per month was positively related to incidents of crop-raiding (F_1,23_ = 12.556, P<0.01), but not by the number of fruits (wild and cultivated combined) available in the farmlands (F_1,23_ = 0.599, P = 0.449), or days when only wild fruits were consumed (F_1,23_ = 0.049, P = 0.828). Thus, orangutans tended to travel further distances on days when they ate cultivated fruits. However, a linear mixed-effect model revealed that no significant single factor influenced the mean day journey length per month of any individual orangutans (crop-raiding days: F_1,30_ = 1.707, P = 0.201; number of fruits (wild and cultivated combined) available: F_1,30_ = 1.529, P = 0.226; sex: F_1,30_ = 1.413, P = 0.244; and, days consuming only wild fruits: F_1,30_ = 0.507, P = 0.482).

## Discussion

Sumatran orangutans are critically endangered, and face major threats from ongoing deforestation, degradation and fragmentation of their rainforest habitats [32]. Recent studies in Borneo have shown that orangutans can maintain healthy population densities in slightly logged forests [33]. However, as most affected forests, and their natural food sources, are being completely replaced by agricultural land uses, especially oil palm plantations, this study answers many basic but fundamentally important questions that were hitherto unknown. We found that orangutans were able to adequately use the habitats of Batang Serangan, but within this the oil palm patches offered few, if any, benefits, as revealed through low levels of both crop-raiding and ranging within them. Furthermore, these patches may actually have been more costly for the male orangutans that moved between the two separated female populations, as the palm leaves are poorly suited for semibrachiator locomotion, and consequently the male orangutans were recorded moving along the ground, here.

The home range sizes of the orangutans in Batang Serangan were small relative to those recorded from other Sumatran studies, but similar to those of wild orangutan populations living in disturbed forest sites at Lokan, Mentoko and Kinabatangan in Borneo (Table 3). Adopting a small home range size may be a response to spatially concentrated wild foods and cultivated fruits. The home range sizes of male orangutans in Batang Serangan were larger than those of females, consistent with other studies which have shown that males travel further in order to maximise their access to receptive females [34]. Orangutans are characterized by a semi-solitary lifestyle, usually living alone with highly overlapping home ranges. Therefore, it is not unexpected that home ranges also overlapped both within and between the sexes at Batang Serangan.

**Table 3:** Orangutan home range size estimates (in ha) from Borneo (B) and Sumatra (S) using minimum convex polygon method.

Source	Study site	Duration (month)	Adult females	Adult males
Singleton & van Schaik (2001)	Suaq Balimbing (S)	52	150 - >850	>2500
Rijksen (1978)	Ketambe (S)	38	150–200	> Females
Unpublished data^1^	Ketambe (S)	48	300–400	> Females
Suzuki (1992)	Mentoko (B)	Several visits	>150	500–700
Mitani (1989)	Mentoko (B)	18	>150	> Females
Rodman (1988)	Mentoko (B)	15	40–60	60–120
Galdikas (1988)	Tanjung Puting (B)	48	350–600	> Females
Horr (1975, 1977)	Lokan (B)	25	65	520
Unpublished data^2^	Kinabatangan (B)	48	180	>225
Unpublished data 3	Tuanan (B)	18	250–300	> Females
Unpublished data^4^	Sabangau (B)	24	250–300	>560
Knott et al. in press	Gunung Palung (B)	103	600	>650
This study	Batang Serangan	24	58–187	330–583

Data table adapted from Singelton et al., 2009 [52] and Utami et al., 2009 [34].

1, Ketambe orangutan project Universitas Nasional Jakarta & Utrecht University Netherlands;

2, Ancrenaz and James;

3, Tuanan orangutan project Universitas Nasional Jakarta & University of Zürich;

4, Morrogh-Bernard.

Numerous studies have linked primate movement patterns with the distribution and abundance of food [29], [35], [36], [37], but few have incorporated crop-raiding behaviour. Those that have reported shifts in home range sizes amongst primate crop raiders include food waste feeding baboons (*Papio cynocephalus*) in Kenya that had greatly reduced home ranges in comparison to those of strictly wild foraging baboons from a neighbouring location [38]. Likewise, smaller home range sizes were recorded among olive baboons (*Papio anubis*) in Kenya that raided crops sporadically during times of low food availability [39]. The smaller than expected home range size of orangutans at Batang Serangan appears to support other findings that crop-raiding primates have smaller home ranges than their non crop-raiding counterparts. Indeed, our results found that raiding cultivated fruits and overall wild and cultivated food availability strongly influenced orangutan ranging strategies, with individuals travelling further across the landscape on days when they were raiding fruits, than when they ate only wild fruits. Other studies have shown that when orangutans encounter an abundance of wild fruits, e.g. during a mast fruiting event, they will tend to greatly reduce their travel distance while staying near the food source [26], [40].

The exceptionally large orangutan home ranges in the peat swamp and lowland forests of Suaq Balimbing have been attributed to the relatively low tree species richness and the coarse grain of the habitat, in which large blocks of distinct habitats are spaced relatively far apart (Table 3; [26]). This habitat would require orangutans to use larger areas to maintain a sufficient diet. However, whilst the Batang Serangan farms also only supports few tree species, the farms are closely spaced, where orangutans might move between patches of different habitats once all the wild and cultivated food resources have been depleted. Therefore, crop-raiding appears to fit many of the predictions of foraging theory [41] and a combination of resource monitoring, diet switching between wild foods and cultivated fruits, and switching from one habitat type ‘patch’ to another, allows orangutans to survive in the Batang Serangan farms.

Daily distances travelled might be expected to be shorter in areas where food is of poor quality and/or patchily distributed, to save energy [31]. However, orangutans in Batang Serangan travelled distances comparable to those reported for males and females from elsewhere [42]. Collectively, the orangutans in Batang Serangan travelled further on crop-raiding days. One plausible explanation for this observation is that, unless eating high energy cultivated fruits, it may be inefficient for orangutans to move longer distances in search of wild fruit due to the energetic costs they would incur [43]. Another plausible explanation may be the homogeneous nature of the farms, in which moving longer distances in a day might not bring orangutans into contact with different habitat types.

Only three previous studies have recorded the true daily linear distance (or nest to nest distance) for orangutans [31], [44], [45], which is an important parameter for investigating how much orangutans deviate on their daily journey. The rather short daily linear distances that have been recorded in Batang Serangan shows that orangutans do not travel in a linear fashion, but more in a circular manner, moving through four or five farms a day in search of food.

The single most important recommendation for conserving orangutans in Batang Serangan is preventing oil palm expansion (i.e. habitat loss) into the closed agroforest system. This would require, first, convincing those smallholder farmers that are planning to do so [21]. Incentive schemes that increase market value of the current crops through improving land productivity, e.g. introducing enhanced rubber germplasm, implementing integrated pest management schemes (including orangutan crop-raiding mitigation) and expanding the number of products extracted from each crop type, are recommended. In this landscape, the value added of having wild, habituated orangutans in the farms could also be increased through developing a community-based nature tourism initiative that generates a local income. Parallel to this, initiatives that engage the oil palm concession holder in sustainable production should be explored, especially through managing this landscape under the Roundtable on Sustainable Palm Oil (RSPO) scheme [46], with an emphasis on High Conservation Value Forest management. Whilst our study reveals the benefits of secondary and degraded forest for orangutans, this should be cautiously interpreted because there are also risks resulting from the increased contact between humans and orangutans. For example, at least two illegal pet infants have been confiscated from Batang Serangan in the last 10 years, meaning that their mothers were almost certainly killed, and other individuals show signs of having been shot at by the local farmers (pers. obs.).

Our study offers important insights for the estimated 75% of Sumatran and Bornean orangutans that live outside of national parks [47] because the majority of these areas have been assigned for oil palm plantations or for commercial logging, which typically begets the former. If future deforestation patterns on Sumatra continue to replace primary and degraded forests with oil palm plantations, we predict that orangutans like those in Batang Serangan are unlikely to survive in the long-term [48]. However, a sustainable solution has been identified by recent studies that have demonstrated the greater economic and biodiversity benefits, including those for orangutans, that can be derived from land use planning and policies that assign forests for avoided deforestation (REDD) projects, rather than for oil palm cultivation [49], [50]. The recent pledge of US$1billion from the Government of Norway to Indonesia in return for reducing deforestation rates is both timely, and welcome, and may avoid imperilling further populations of orangutans [51].
